# Evidence for Narrow Transfer after Short-Term Cognitive Training in Older Adults

**DOI:** 10.3389/fnagi.2017.00041

**Published:** 2017-02-28

**Authors:** Dustin J. Souders, Walter R. Boot, Kenneth Blocker, Thomas Vitale, Nelson A. Roque, Neil Charness

**Affiliations:** Department of Psychology, Florida State UniversityTallahassee, FL, USA

**Keywords:** cognitive training intervention, reasoning ability, video games, transfer of training, cognitive aging

## Abstract

The degree to which “brain training” can improve general cognition, resulting in improved performance on tasks dissimilar from the trained tasks (transfer of training), is a controversial topic. Here, we tested the degree to which cognitive training, in the form of gamified training activities that have demonstrated some degree of success in the past, might result in broad transfer. Sixty older adults were randomly assigned to a gamified cognitive training intervention or to an active control condition that involved playing word and number puzzle games. Participants were provided with tablet computers and asked to engage in their assigned training for 30 45-min training sessions over the course of 1 month. Although intervention adherence was acceptable, little evidence for transfer was observed except for the performance of one task that most resembled the gamified cognitive training: There was a trend for greater improvement on a version of the corsi block tapping task for the cognitive training group relative to the control group. This task was very similar to one of the training games. Results suggest that participants were learning specific skills and strategies from game training that influenced their performance on a similar task. However, even this near-transfer effect was weak. Although the results were not positive with respect to broad transfer of training, longer duration studies with larger samples and the addition of a retention period are necessary before the benefit of this specific intervention can be ruled out.

## Introduction

Increases in life expectancy, along with decreasing fertility rates, have led to older adults making up a larger proportion of the global population than ever before (i.e., Population Aging; United Nations, 2015). This trend is significant because age-related changes in cognition can threaten the ability of older adults to live independently, and the societal cost of supporting an increasing number of older adults may be quite large. Considering this demographic trend and its implications, exploring methods to stave-off age-related cognitive decline is important, and has been of increasing interest to the scientific community (e.g., Hertzog et al., 2008).

Greenwood and Parasuraman (2010) hypothesized that successful cognitive aging involves the interaction between neuronal plasticity (i.e., structural brain changes on the cellular level stimulated by experience) and cognitive plasticity (i.e., changes in cognitive strategy). This would be the case if (1) the normal mechanisms of neuronal plasticity are sustained into old age, (2) exposure to novelty in terms of new experiences continues to drive changes in neuronal plasticity, and (3) neural integrity is upheld by beneficial diet, exercise, and other factors. There are a number of studies that support the idea that older adults' brains retain plasticity (i.e., the ability to adapt or benefit from experiences), which suggests that by making healthy lifestyle choices (e.g., balanced diet, regular exercise) and/or engaging in cognitively demanding activities, older adults can maintain a high level of cognitive functioning (reviewed in detail in Greenwood and Parasuraman, 2012). In other words, new learning can result in cognitive plasticity, which encourages neuroplasticity, which then supports additional learning. This theoretical account is consistent with claims made by the proponents of brain training programs.

The potential benefits of cognitive training interventions aimed at averting or reducing cognitive decline in old age have led to the emergence of commercial programs with the aim of improving cognition through game-like tasks. Brain training is currently a billion dollar industry (Commercialising Neuroscience: Brain Sells, 2013; Sharp Brains, 2013). However, the degree to which brain training (especially commercially available brain training programs) is effective, remains controversial. Currently available data have spawned dissenting “consensus” statements, one arguing against the efficacy of brain training with respect to meaningfully improving cognition (A consensus on the brain training industry from the scientific community, 2014) and one arguing for it (Cognitive Training Data, 2014). These opposing statements with hundreds of academic signatories highlight that the effectiveness of commercial cognitive training for older adults remains unclear. Many of the promised benefits of brain training are vague and the evidence that brain training companies point to in support of their products' effectiveness is often flawed (Simons et al., 2016).

Cognitive training as a means of combatting age-related cognitive decline hinges on the notion that training specific cognitive functions that support the performance of a variety of tasks (e.g., working memory) can lead to improvements on many tasks beyond the trained one (i.e., far transfer). Jonides (2004) has argued that transfer from a trained to an untrained task occurs when the two tasks share processing components and activate overlapping brain regions. Many efforts to induce far transfer have focused on training working memory, due to its integral and ubiquitous role in many other cognitive and everyday tasks, with pre- to post-training increases sometimes observed in younger adults' executive functions and reasoning or memory (e.g., Jaeggi et al., 2008; Au et al., 2015). However, these findings are far from uncontroversial (e.g., Morrison and Chein, 2011; Shipstead et al., 2012; Melby-Lervåg and Hulme, 2013; Melby-Lervåg et al., 2016).

In some cases, transfer to untrained cognitive tasks that recruit working memory in older adults seems more limited relative to younger adults, so it is important to examine the effect of training in both populations. Dahlin et al. (2008) randomly assigned older and younger adults to a group that received memory-updating training or to a control group that received no training. Transfer to tasks involving perceptual speed, working memory, episodic memory, verbal fluency, and reasoning was assessed after 5 weeks of training and in an 18-month follow-up post-training. Results showed that both younger and older adults that received the training improved on the trained tasks, and these benefits were still evident at the 18-month follow-up. Younger participants also showed transfer to a 3-back task, which required updating similar to the trained task but was not trained. However, older participants in this study did not show similar transfer. Further, in younger adults, no other transfer was observed to tasks of fluency or reasoning, lending support to the notion that transfer is only possible when untrained tasks utilize similar processing components (in this case, the striatum) as the trained tasks.

It is reasonable to expect improvement on trained cognitive tasks after undergoing training, and studies with older adults are consistent with this expectation (e.g., Ball et al., 2002; Willis et al., 2006). However, examples of successful far transfer from cognitive training to everyday functioning are rare. Follow-up studies from the ACTIVE trial and other studies using speed of processing training are some of the most widely cited examples in the literature of far transfer to everyday functioning, attributed to older adults' participation in cognitive training. Participants that had taken part in either the speed of processing or reasoning training in the ACTIVE trial were reported to have lower rates of at-fault collision involvement in the 6 years following their involvement in the study (Ball et al., 2010), though higher rates of not-at-fault collisions with no overall collision benefit observed. Other studies that have used this speed of processing training in older adults have found that participants receiving training have reported less driving difficulty, more driving time, and longer driving distances than controls (Edwards et al., 2009b), as well as fewer driving cessations after a 3-year follow-up period (Edwards et al., 2009a). Still, overall, the literature is mixed, and the ACTIVE trial and various follow up studies, when examined closely, seem to provide only limited evidence for the benefits of training (Simons et al., 2016).

Due to the precarious support for cognitive training's benefit for older adults, it is important to compare observed cognitive benefits to those of a strong, active control group with surface plausibility. Mentally stimulating activities, such as word or number puzzles, have long been commonly thought to stave off cognitive decline, though evidence for their benefit is lacking. The current study investigated the cognitive effects in older adults using a gamified cognitive training suite (Mind Frontiers) compared to those in an active control group that played similarly-delivered word and number puzzles (crossword, word search, and Sudoku) that were believed by participants to be cognitively beneficial (Boot et al., 2016).

## Methods

### Participants

Our goal was to obtain a sample 60 older adults (age 65+) to be randomly and evenly distributed between two conditions (intervention and control; *N* = 30 per group). Due to the attrition of 18 participants, 78 older adults were recruited from the Tallahassee, Florida region, the majority of whom were recruited via the lab's participant database. Six participants dropped out of the study from the control group, eight from the intervention group, and four before random assignment. All participants were prescreened to assess basic demographic information as well as to ensure that they met the criteria necessary to qualify for the study (e.g., English fluency, no limiting physical and/or sensory conditions). To ensure that potential participants were cognitively intact, the short portable mental status questionnaire (Pfeiffer, 1975) was used, as well as the logical memory subscale of the Wechsler Memory Scale (age-adjusted; Wechsler, 1997). Descriptive information concerning the sample is available in Table 1. This study was carried out in accordance with the recommendations of the Belmont Report with written informed consent from all subjects. All subjects gave written informed consent in accordance with the Declaration of Helsinki. The protocol was approved by Florida State University's Human Subjects Committee. Participants were compensated $20 for the initial lab visit, $60 for completing the at-home training, and $20 after returning the training materials and finishing the post-training cognitive assessment, totaling $100.

**Table 1:** **Descriptive information regarding the sample of older adults that participated in the study**.

**Variable**	**Overall *N* = 60**	**Mind frontiers *n* = 30**	**Control *n* = 30**
**AGE IN YEARS MEAN (*****SD*****)**			
	72.35 (5.20)	72.27 (4.88)	72.43 (5.58)
**GENDER** ***N*** **(%)**			
Female	34 (56.7%)	17 (56.7%)	17 (56.7%)
Male	26 (43.3%)	13 (43.3%)	13 (43.3%)
**ETHNICITY** ***N*** **(%)**			
African-American	3 (5%)	1 (3.3%)	2 (6.7%)
Caucasian	55 (91.7%)	29 (96.7%)	26 (86.7%)
Hispanic	2 (3.3%)	0 (0%)	2 (6.7%)
**EDUCATION** ***N*** **(%)**			
High school/some college	20 (33.3%)	12 (40.0%)	8 (26.7%)
College degree	40 (66.7%)	18 (60.0%)	22 (73.3%)

### Study design

Once eligibility was confirmed, participants were randomly assigned to either the intervention or control conditions and completed a battery of cognitive tests to assess baseline cognitive functioning. Participants then attended a 2-h training session, which involved a tutorial on how to use the provided tablet (10 inch Acer Iconia A700), as well as how to play the games they were assigned per their condition. Over the course of 1 month, participants in both groups were asked to play three games per day (including weekends) for 15 min each, totaling 45 min of playtime each session. Journals were given to participants to record their playtime.

Participants in the intervention condition were provided tablets with the Mind Frontiers application preinstalled on their system. Mind Frontiers is a Western-themed game hub comprised of seven gamified cognitive tasks modified to improve the tasks' aesthetics, as well as to include motivating feedback to encourage participants to continue playing the games (see Baniqued et al., 2015 for more details). These gamified tasks were designed to exercise inductive reasoning, planning, spatial reasoning ability, speed of processing, task switching, and working memory updating. For an overview of the games included in Mind Frontiers, see Table 2. Participants played a subset of the seven games that varied each day to ensure that the same games were not played in consecutive sessions. In comparison, participants assigned to the control condition were tasked with playing three common puzzle games each day (crossword, Sudoku, and word search). In both conditions, tablets were locked-down so that participants could not use the tablet for any other purpose. After 1 month of playtime, participants returned to the laboratory with the tablets and journals and completed the post-training cognitive battery.

**Table 2:** **A brief description of each of the seven games included in the Mind Frontiers application**.

**Mind Frontiers Game**	**Description**
Ante Up	Players are shown cards organized in a certain pattern and must replicate this pattern over the course of a specified number of moves with the cards they are provided. This game exercises planning ability and is based on the Tower of London test (Shallice, 1982).
Irrigator	Players are tasked with building a water pipeline from a well to various targets before time runs out using provided pipe pieces that change with each turn. As players progress, various obstacles must be avoided to reach the target. This game challenges visuospatial processing and is similar to a training task previously used by Mackey et al. (2011).
Pen ‘Em Up	Players must sort objects dropped from a UFO into two pens by swiping either left or right based on specific criteria provided at the start. The sorting criteria varies based upon the objects' characteristics (e.g., trees, farm animals) or style (e.g., plain, striped). This task-switching game is based on the training developed by Karbach and Kray (2009).
Riding Shotgun	Players are riding in a horse drawn wagon, and the scene in front of the wagon contains a grid of tiles that could light up one at a time. The player must remember the sequence in which tiles of the grid are illuminated. They must then replicate the pattern in the correct order. This task taps visuospatial memory and is similar to the training provided by Klingberg et al. (2002).
Safe Cracker	Players are tasked with cracking codes to open various safes and collect the money inside before the time runs out. Stimuli are series of letters, numbers, months, etc., and the player must determine the next item in the sequence. This game is intended to exercise inductive reasoning skills and is similar to the training described by Willis and Schaie (1986).
Sentry Duty	Players are tasked with remembering the sequence in which sentries outside of a fort wall lift a lantern and say a word. They must decide whether the location and word of the current sentry matches that of the sentry *N* turns prior. This dual n-back game challenges working memory and is similar to the training task developed by Jaeggi et al. (2008).
Supply Run	Players adopt the role of a merchant traveling through a town. Townspeople request items along the way. The player must remember the last item requested from each of the provided categories so they may be purchased at a town store at the end of the trip. This working memory game is similar to the training used by Dahlin et al. (2008).

### Measures

#### Cognitive battery

A cognitive battery was administered before and after the at-home training to establish a baseline performance level and to measure change in performance as a function of training. The battery was comprised of nine computer and paper and pencil-based cognitive and perceptual assessments^1^.

#### Reasoning ability

Four computerized tests were used to assess reasoning ability: form boards, letter sets, paper folding, and Raven's Advanced Progressive Matrices.

##### Form boards

For each problem, participants were shown a target shape and had to select which of the presented shapes would fill the target shape exactly (Ekstrom et al., 1976). This task primary tapped visuospatial reasoning. Two alternative forms were presented to participants before and after training (counterbalanced). Participants were allotted 8 min to complete as many problems correctly as possible out of a total of 24 problems (primary measure).

##### Letter sets

For each problem, participants were presented with a set of five strings of letters (Ekstrom et al., 1976). Participants identified the one letter set that did not conform to the same rule as the others. This task served as a measure of inductive reasoning. Participants were allowed 10 min to complete as many problems as possible out of 15. Two alternative forms were presented to participants before and after training (counterbalanced). The primary measure of performance was the number of correctly solved problems.

##### Paper folding

For each problem, participants were shown a folded piece of paper with a hole punched through it (Ekstrom et al., 1976). The task of the participant was to identify the pattern that would result when the paper was unfolded. This represented a measure of spatial reasoning ability. Participants were given 10 min to solve a maximum of 12 problems. Two alternative forms were counterbalanced across pre- and post-testing. The primary measure of performance was the number of correctly solved problems.

##### Ravens matrices

Each problem presented participants with a complex pattern (in the form of a 3 × 3 matrix; Raven, 1962). The task of the participant was to identify the option that would complete the missing piece from the pattern. This was a measure of fluid intelligence. Participants were given up to 10 min to solve a maximum of 18 problems. Two alternative forms were counterbalanced across pre- and post-testing. The number of correctly solved problems served as the primary measure of performance.

#### Processing speed

Two measures of processing speed were administered: pattern comparison (paper and pencil) and simple/complex response time (computer test).

##### Pattern comparison

Participants viewed several pairs of line figures on each page and had to write “S” or “D” (for “same” or “different”) between them depending on whether the figures were identical or not (Salthouse and Babcock, 1991). Participants completed two pages each assessment, with 30 s allowed for each page. Two parallel forms were administered before and after training, and form order was counterbalanced. Total number of correct responses within the allotted time was used as the primary measure of performance.

##### Simple/choice reaction time

This task was similar to the one administered previously by Boot et al. (2013). In two blocks of trials, participants saw a green square appear at the center of the screen and had to push a key as quickly as possible when it appeared (30 trials each block). In another block of trials, the box appeared to the right or left side of the screen and participants pushed one of two buttons to indicate its location (60 trials). Average speed of accurate trials was used as the primary measure of performance.

#### Memory

One computerized test was used to assess memory.

##### Corsi block tapping

This task (similar to Corsi, 1972) was run using PEBL (Mueller and Piper, 2014). Participants viewed a spatial array of nine blue squares on the screen. These squares changed one at a time from blue to yellow, then back to blue, in a randomized sequence. Participants were asked to replicate the observed sequence using the mouse and then click the done button at the bottom of the screen. Each participant completed three unrecorded practice trials and was given feedback in order to become familiar with the task. The recorded task's sequence started with two squares and each participant completed two trials of each sequence length before the length increased by one. The sequence increased by one whenever the participant correctly demonstrated at least one of the two sequences at that sequence length, and the task ended if the participant failed both of the sequences at a given length. The primary measure of this task was a span measure based on the length of the sequence when the task ended.

#### Executive control

A task-switching paradigm (computerized) and Trails B (paper and pencil) were used to assess executive control.

##### Task-Switching

This task was similar to the one used by Boot et al. (2013). Participants viewed digits that appeared one at a time at the center of the screen for 2.5 s each and had to judge whether each digit was high or low (above or below 5), or whether it was odd or even depending on the color of the square surrounding the digit (blue or pink). A blue square indicated participants had to judge whether the number was high or low, while a pink square indicated that participants had to judge whether the digit was odd or even. The digits 1 through 9 were randomly presented, with the exception that the digit 5 was never used. The “z” key was used to indicate either low or odd while the “/” key was used to indicate high or even. Participants completed four blocks of 15 trials each in which they only had to perform one task or the other. Then they completed a version of the task in which the color of the background was randomized, meaning they often had to switch from one task to the other. After 15 practice dual-task trials, participants completed 160 real trials. Switch cost, or the decrement involved in having to switch from one task to another, was used as the primary measure of task-switching. This was calculated by comparing the average performance (accurate response time) of single task blocks of trials to the dual-task block.

##### Trails B (controlling for Trails A)

In this task, participants were presented with a sheet of paper containing numbers and letters (Reitan, 1955). Participants were asked to connect the numbers and letters in sequential order, alternating between numbers and letters (1, A, 2, B, etc.). Completion time was the primary measure of performance. Completion time of Trails A, in which participants performed the same task but did not have to switch between numbers and letters, was subtracted from Trails B completion time to provide a measure of switch cost.

## Results

**Figure 1:**
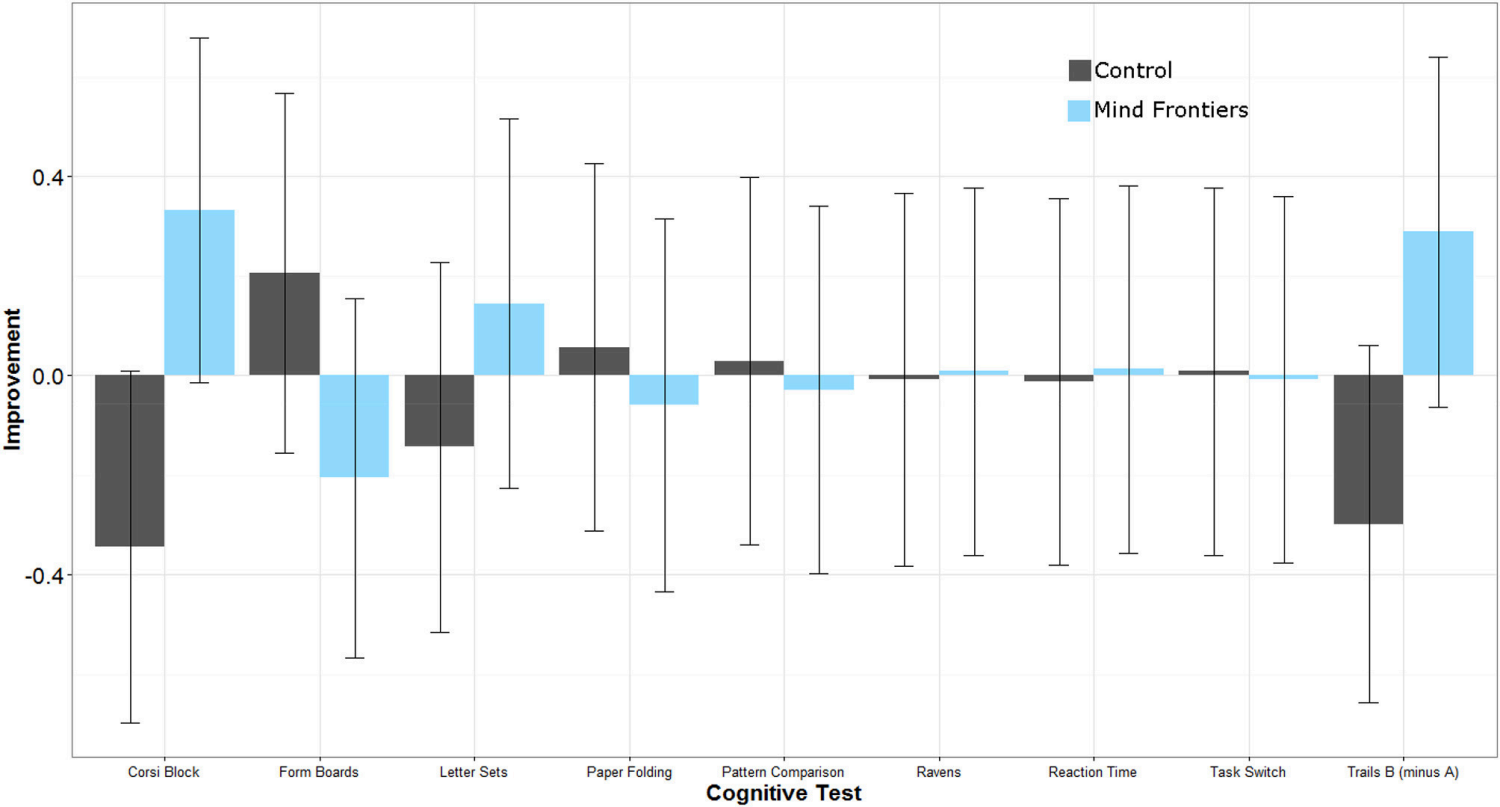
**Standardized improvement scores (larger scores represent greater improvement) for all cognitive measures as a function of condition**. Error bars represent 95% Confidence Intervals.

Intervention adherence was acceptable in that participants engaged in, on average, more than 70% of their assigned training sessions according to their journals (*M* = 22 sessions, *SD* = 9.0 vs. *M* = 23 sessions, *SD* = 7.7 for the control and intervention groups, respectively). To help interpret results in light of potential placebo effects, participants' expectations for improvement were assessed after training. These data are reported elsewhere, so will not be discussed in detail, but in general participants in each group either expected a similar amount of improvement as a result of training, or expected the control condition to improve cognition more (Boot et al., 2016). Any differential improvement by the intervention group is thus unlikely due to a placebo effect. Next we turn to potential changes in performance on tasks in the cognitive battery. Our general analysis approach was to explore whether groups differed on their post-training performance controlling for pre-training scores. Scores for all measures are reported in Table 3 and standardized (*z*-score) improvement scores are represented in Figure 1. Note that the degrees of freedom fluctuate slightly in the reported analyses due to occasional missing data.

**Table 3:** **Means and 95% CIs for all measures**.

**Test**	**Measure**	**Group**	**n**	**Pretest**	**Posttest**	**Difference**	**ANCOVA Group Effect**
Form Boards	#Correct	Control	30	5.20 [4.07, 6.33]	6.20 [4.88, 7.52]	1.00 [−0.38, 2.38]	*p* = 0.09, $\eta_{p}^{2}$ = 0.05
		MF	30	5.40 [3.93, 6.87]	5.00 [3.82, 6.18]	−0.40 [−1.51, 0.71]	
Letter Sets	#Correct	Control	29	9.17 [8.37, 9.98]	9.17 [8.18, 10.17]	0.00 [−0.87, 0.87]	*p* = 0.19, $\eta_{p}^{2}$ = 0.03
		MF	29	9.17 [7.91, 10.43]	9.86 [9.00, 10.73]	0.69 [−0.25, 1.63]	
Paper Folding	#Correct	Control	30	4.40 [ 3.46, 5.34]	4.77 [3.92, 5.61]	0.37 [−0.56, 1.38]	*p* = .59, $\eta_{p}^{2}$ = 0.005
		MF	29	4.37 [3.54, 5.22]	4.45 [3.50, 5.39]	0.07 [−0.86, 1.00]	
Ravens	#Correct	Control	29	3.62 [2.69, 4.55]	3.59 [2.89, 4.29]	−0.03 [−0.72, 0.65]	*p* = 0.96, $\eta_{p}^{2}$ < 0.001
		MF	30	3.60 [2.62, 4.58]	3.60 [2.78, 4.41]	0.00 [−0.98, 0.98]	
Reaction Time	RT (ms)	Control	30	396 [378, 413]	385 [366, 403]	10 [−3, 23]	*p* = 0.46, $\eta_{p}^{2}$ < 0.01
		MF	30	373 [357, 388]	361 [345, 378]	11 [−3, 26]	
Pattern Comparison	#Correct	Control	30	24.40 [21.82, 26.98]	24.17 [21.45, 26.88]	−0.23 [−3.81, 3.34]	*p* = 0.57, $\eta_{p}^{2}$ < 0.01
		MF	30	26.07 [23.84, 28.30]	25.37 [23.45, 27.28]	−0.70 [−3.17, 1.76]	
Task Switch	Switch Cost (ms)	Control	30	248 [135, 362]	237 [177, 297]	12 [−100, 123]	*p* = 0.22, $\eta_{p}^{2}$ = 0.03
		MF	30	321 [221, 422]	314 [232, 397]	7 [−86, 100]	
Trails B (Minus A)	Switch Cost (s)	Control	29	41 [32, 50]	48 [37, 60]	−7 [−16, 2]	*p* = 0.07, $\eta_{p}^{2}$ = 0.06
		MF	30	50 [41, 59]	43 [36, 49]	7 [−2, 16]	
Corsi Block	Span	Control	29	4.50 [4.20, 4.80]	4.59 [4.29, 4.88]	0.09 [−0.15, 0.32]	*p* = 0.009, $\eta_{p}^{2}$ = 0.16
		MF	30	4.38 [4.13, 4.64]	4.90 [4.64, 5.16]	0.52 [0.29, 0.74]	

*P-alues and effect sizes represent the effect of group on posttest performance controlling for pretest performance. Difference scores were computed for all measures such that higher scores represent greater improvement. Effect sizes are reported as partial eta-squared ($\eta_{p}^{2}$). Cohen (1969) recommendation for interpretation are ~0.01 for small, 0.06 for medium, and 0.14 for large effects*.

### Reasoning ability

#### Form boards

Time 2 (post-training) scores were entered into an ANCOVA with Time 1 (baseline) scores as a covariate and group (Mind Frontiers vs. Control) as a between-participant variable. This analysis revealed a significant effect of the Time 1 covariate [*F*_(1, 57)_ = 21.65, *p* < 0.001, η_*p*_^2^ = 0.28], but no effect of group [*F*_(1, 57)_ = 3.04, *p* = 0.09, η_*p*_^2^ = 0.05]. Adjusted marginal means revealed numerically better post-training performance for the control group relative to the intervention group (*M*_*adj*_ = 6.25, *SE* = 0.53 vs. *M*_*adj*_ = 4.95, *SE* = 0.53 for the control and intervention groups, respectively).

#### Letter sets

Time 2 (post-training) scores were entered into an ANCOVA with Time 1 (baseline) scores as a covariate and group (Mind Frontiers vs. Control) as a between-participant variable. This analysis revealed a significant effect of the Time 1 covariate [*F*_(1, 55)_ = 29.55, *p* < 0.001, η_*p*_^2^ = 0.35], but no effect of group [*F*_(1, 55)_ = 1.73, *p* = 0.19, η_*p*_^2^ = 0.03]. Adjusted marginal means were similar for the control group and the intervention group (*M*_*adj*_ = 9.17, *SE* = 0.37 vs. *M*_*adj*_ = 9.86, *SE* = 0.37 for the control and intervention groups, respectively).

#### Paper folding

Time 2 (post-training) scores were entered into an ANCOVA with Time 1 (baseline) scores as a covariate and group (Mind Frontiers vs. Control) as a between-participant variable. This analysis revealed a significant effect of the Time 1 covariate [*F*_(1, 56)_ = 11.02, *p* < 0.01, η_*p*_^2^ = 0.16], but no effect of group [*F*_(1, 56)_ = 0.30, *p* = 0.59, η_*p*_^2^ = 0.005]. Adjusted marginal means were similar for the control group and the intervention group (*M*_*adj*_ = 4.76, *SE* = 0.40 vs. *M*_*adj*_ = 4.45, *SE* = 0.41 for the control and intervention groups, respectively).

#### Ravens

Time 2 (post-training) scores were entered into an ANCOVA with Time 1 (baseline) scores as a covariate and group (Mind Frontiers vs. Control) as a between-participant variable. This analysis revealed a significant effect of the Time 1 covariate [*F*_(1, 56)_ = 21.89, *p* < 0.001, η_*p*_^2^ = 0.28], but no effect of group [*F*_(1, 56)_ = 0.002, *p* = 0.96, η_*p*_^2^ < 0.001]. Adjusted marginal means were similar for the control group and the intervention group (*M*_*adj*_ = 3.58, *SE* = 0.32 vs. *M*_*adj*_ = 3.60, *SE* = 0.32 for the control and intervention groups, respectively).

### Processing speed

#### Simple/choice reaction time

An aggregate speed measure was created by averaging simple and choice reaction time conditions. Time 2 (post-training) scores were entered into an ANCOVA with Time 1 (baseline) scores as a covariate and group (Mind Frontiers vs. Control) as a between-participant variable. This analysis revealed a significant effect of the Time 1 covariate [*F*_(1, 57)_ = 49.52, *p* < 0.001, η_*p*_^2^ = 0.47], but no effect of group [*F*_(1, 57)_ = 0.55, *p* = 0.46, η_*p*_^2^ < 0.01]. Adjusted marginal means were similar for the control group and the intervention group (*M*_*adj*_ = 376 ms, *SE* = 6.58 vs. *M*_*adj*_ = 370 ms, *SE* = 6.58 for the control and intervention groups, respectively).

#### Pattern comparison

Number of correct responses per allocated time served as the primary measure of performance. Time 2 (post-training) scores were entered into an ANCOVA with Time 1 (baseline) scores as a covariate and group (Mind Frontiers vs. Control) as a between-participant variable. This analysis revealed no significant effect of the Time 1 covariate [*F*_(1, 57)_ = 1.66, *p* = 0.20, η_*p*_^2^ = 0.02] and no effect of group [*F*_(1, 57)_ = 0.32, *p* = 0.57, η_*p*_^2^ < 0.01]. Adjusted marginal means were similar for the control group and the intervention group (*M*_*adj*_ = 24, *SE* = 1.15 vs. *M*_*adj*_ = 25, *SE* = 1.15 for the control and intervention groups, respectively).

### Executive control

#### Task-switching

Switch cost in terms of response speed was chosen as the primary measure of performance for this task. Time 2 (post-training) scores were entered into an ANCOVA with Time 1 (baseline) scores as a covariate and group (Mind Frontiers vs. Control) as a between-participant variable. This analysis revealed a significant effect of the Time 1 covariate [*F*_(1, 57)_ = 10.42, *p* < 0.01, η_*p*_^2^ = 0.16], but no effect of group [*F*_(1, 57)_ = 1.56, *p* = 0.22, η_*p*_^2^ = 0.03]. Adjusted marginal means were similar for the control group and the intervention group (*M*_*adj*_ = 246 ms, *SE* = 32.81 vs. *M*_*adj*_ = 305 ms, *SE* = 32.81 for the control and intervention groups, respectively).

#### Trails B (minus Trails A)

Trails B completion time minus Trails A time served as a measure of executive control. Time 2 (post-training) scores were entered into an ANCOVA with Time 1 (baseline) scores as a covariate and group (Mind Frontiers vs. Control) as a between-participant variable. This analysis revealed a significant effect of the Time 1 covariate [*F*_(1, 56)_ = 20.81, *p* < 0.001, η_*p*_^2^ = 0.27], but no effect of group [*F*_(1, 56)_ = 3.42, *p* = 0.07, η_*p*_^2^ = 0.06]. Adjusted marginal means revealed numerically better post-training performance (smaller cost for Trails B relative to A) for the intervention group relative to the intervention group (*M*_*adj*_ = 51 s, *SE* = 3.97 vs. *M*_*adj*_ = 40 s, *SE* = 3.91 for the control and intervention groups, respectively).

### Memory

#### Corsi block tapping

Memory span served as the primary measure of performance. Time 2 (post-training) scores were entered into an ANCOVA with Time 1 (baseline) scores as a covariate and group (Mind Frontiers vs. Control) as a between-participant variable. This analysis revealed a significant effect of the Time 1 covariate [*F*_(1, 56)_ = 44.48, *p* < 0.001, η_*p*_^2^ = 0.44], and a trend for an effect of group [*F*_(1, 56)_ = 7.31, *p* = 0.009, η_*p*_^2^ = 0.16], with better performance for the intervention group compared to the control group (*M*_*adj*_ = 4.55, *SE* = 0.103 vs. *M*_*adj*_ = 4.94, *SE* = 0.101 for the control and intervention groups, respectively). While these data are consistent with a benefit, they should be considered in light of the number of analyses conducted. This effect would not be significant under a conservative Bonferroni correction (alpha 0.05/9 tests = 0.0056).

### Game improvement

**Figure 2:**
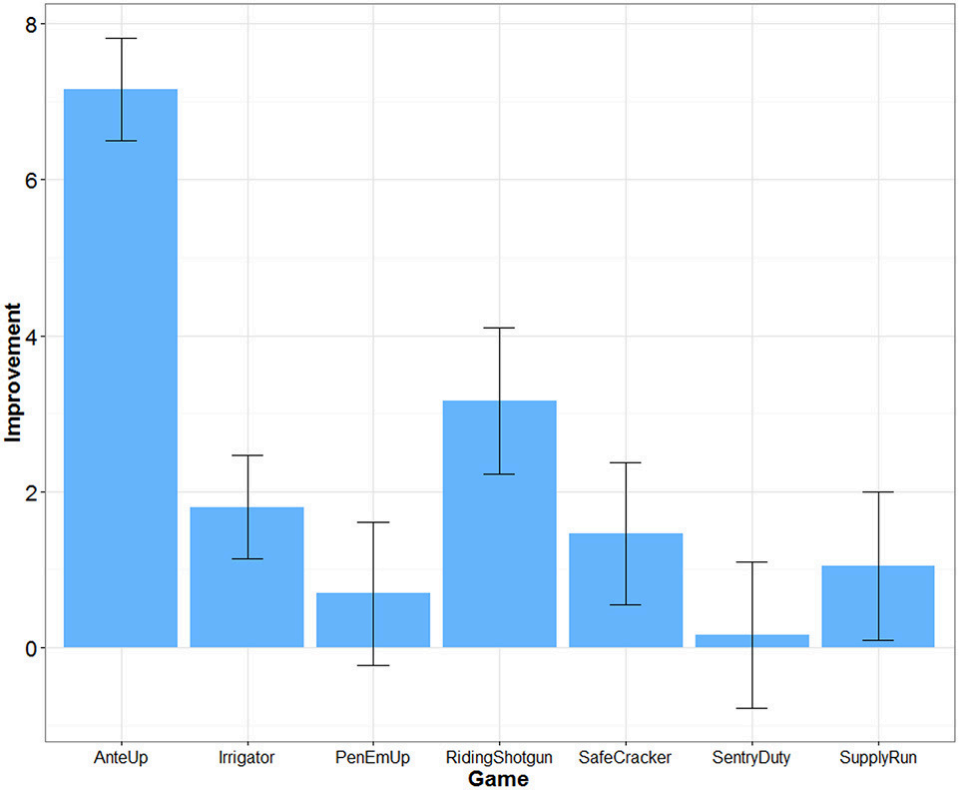
**Improvement in game difficulty level as a function of game**. Error bars represent 95% Confidence Intervals.

The difficulty of each game was adjusted over time depending on participants' performance. Transfer effects are unlikely if participants did not improve their game performance. An algorithm within each game dynamically adjusted difficulty based on successful game performance each time a game was played. For example, for *Riding Shotgun*, correctly remembering a sequence increased the difficulty level and added one more item to the sequence to be remembered. Incorrectly recalling a sequence decreased the difficulty level and removed one item from the sequence to be remembered. We were able to extract game performance data for 25 participants in the Mind Frontiers condition. Figure 2 depicts the average increase in game difficulty level over the course of training for each game. What was observed was a mixed pattern of improvement. Little improvement was observed for *Sentry Duty*, the game closest to n-back training which has been argued to improve fluid intelligence. However, improvement was observed for the *Riding Shotgun* game which is similar to the corsi block tapping task. A trend for improvement was observed for this outcome measure, though improvement was not significant after correcting for the number of outcome measures tested. Baniqued et al. (2015) similarly found that participants only improved to a small degree on *Sentry Duty* and *Supply Run* compared to other games in a study involving a younger adult sample. Across games, it is unclear whether differences in improvement relate to the demands of the game or scoring algorithm used by the Mind Frontiers software package.

## Discussion

In general, little evidence of transfer was observed in the current study. The only measure that hinted at a benefit for the intervention group relative to the control group was the corsi block tapping task. Within the Mind Frontiers game suite, the *Riding Shotgun* game was essentially a gamified version of this outcome task. Even though a dual n-back training component was part of the current intervention, and previous studies have linked this type of intervention to improved reasoning ability (especially with respect to matrix reasoning tasks, e.g., Jaeggi et al., 2008), no benefits were observed as a result of the intervention. Evidence was largely consistent with a recent review of the literature in that the strongest evidence was for near rather than far transfer of training (Simons et al., 2016). Even this effect, though, was ambiguous given the number of measures collected and the possibility of Type I error.

All studies examining brain training effects need to be considered in light of potential methodological and statistical shortcomings (Simons et al., 2016). Some of these shortcomings may overestimate the potential of brain training and others may underestimate it. Next we present a discussion of these issues.

To guard against the effect of experimenter degrees of freedom (flexibility in the way analyses can be conducted that increase the likelihood of false-positive results; Simmons et al., 2011), it is now generally recommended that studies be preregistered. The current study was not preregistered, but our lab has made a commitment to preregister future cognitive intervention studies based on current recommendations. When study design and analysis approaches are not preregistered, positive findings here and elsewhere provide less convincing evidence in favor of brain training effects. In the absence of preregistration it is unclear whether Type I error was appropriately controlled for. Despite the absence of preregistration, little evidence of transfer was observed. Thus, Type I error control was unlikely to be a large problem here with respect to overestimating the degree of transfer.

To guard against placebo effects that may overestimate transfer effects, studies should include a strong active control condition. The current study had an active control condition featuring games that were not expected to tap the same perceptual and cognitive abilities exercised by the Mind Frontiers game. Further, expectation checks should be implemented to ensure that differential improvement of the intervention group isn't linked to greater expectations for improvement (with differential effort exerted post training for the task with greater expectations). Expectation checks were included in the current study, and it was found that any differential improvement of the intervention group (even though little evidence of transfer was observed) would be unlikely due to a placebo effect (Boot et al., 2016). However, this analysis indicated that the intervention and control groups were not perfectly matched; participants in the Mind Frontiers group actually expected *less* improvement with respect to changes in vision and response time. It is conceivable that the greater expectations of the control group may have masked transfer produced by the intervention (see Foroughi et al., 2016; for evidence that expectations can influence cognitive task performance).

Statistical power should always be considered as well when evaluating the effect of cognitive training interventions. With approximately 30 participants in each group for reported analyses and using an ANCOVA approach, the current study was powered only to detect large effects (*f* = 0.40) with a probability of about 0.80 using an alpha level of 0.05. This means that subtle effects may have gone undetected.

Dosage, retention, and training gains are important issues as well. Had participants adhered perfectly, they would have completed 22.5 h of training. While this is a reasonable dosage in comparison to many studies (e.g., the ACTIVE trial), cognitive intervention effects may require similar engagement over many months or years to provide protection against cognitive decline. Almost no studies to date have examined the effect of long-term engagement in cognitive training (see Requena et al., 2016; for an exception). Studies such as ours, typical of the field, test whether or not there may be a “quick fix” provided by cognitive training. In addition to overall dosage, it may be important to consider dosage of each game within the Mind Frontiers suite. With seven total games, participants were asked to play about 3 h of each game over the course of 1 month. If some of these games are more effective than others at producing near and far transfer, the training schedule of our study may underestimate transfer. Of particular note is the lack of improvement for some games (Figure 2). Although game timing parameters were adjusted to be more appropriate for older adult participants, participants appeared to struggle with making progress within some games, especially *Sentry Duty* and *Pen ‘Em Up*. Given the difficulty and complexity of these two game in particular, it is possible that improved game instructions and training might help participants make more progress.

Finally, our study examined transfer immediately after training. While it is reasonable to assume effects would be largest immediately after training, others have suggested that cognitive protection provided by cognitive training may not be observed until cognition begins to show steeper declines. Our study did not assess performance at later time points (as the ACTIVE trial has nicely done), so we cannot rule out such “sleeper effects” of cognitive training.

Which particular mechanisms are responsible for the benefits of cognitive training, and the question of whether broad transfer from cognitive training is even possible, are currently controversial topics. No one study provides a definitive answer and evidence needs to be evaluated with respect to the strength of a study's design and analysis approach. The current study contributes to the idea that there are no short-term, easy methods to boost cognitive performance in older adults. Whether other cognitive training interventions or longer-term interventions can produce broad transfer and improve the performance of everyday tasks important for independence (e.g., driving, financial management) remains to be seen.

## Author contributions

WB, DS, and NC designed the study. KB, TV, and DS supervised data collection and data management. NR assisted with data processing and analysis. DS and WB completed the first draft of the manuscript, and all authors were involved in the editing process.

### Conflict of interest statement

Aptima, Inc., designer of the Mind Frontiers software package, provided technical support for the reported project at no cost. The authors declare that the research was conducted in the absence of any commercial or financial relationships that could be construed as a potential conflict of interest.

## Footnotes

^1^The battery originally contained 10 measures, but due to a programming error data from an n-back working memory test could not be analyzed.
